# Need for and use of contraception by women before and during COVID-19 in four sub-Saharan African geographies: results from population-based national or regional cohort surveys

**DOI:** 10.1016/S2214-109X(21)00105-4

**Published:** 2021-05-18

**Authors:** Shannon N Wood, Celia Karp, Funmilola OlaOlorun, Akilimali Z Pierre, Georges Guiella, Peter Gichangi, Linnea A Zimmerman, Philip Anglewicz, Elizabeth Larson, Caroline Moreau

**Affiliations:** a Department of Population, Family and Reproductive Health, Johns Hopkins Bloomberg School of Public Health, Baltimore, MD, USA; b College of Medicine, University of Ibadan, Ibadan, Nigeria; c Kinshasa School of Public Health, Kinshasa, DR Congo; d Institut Supérieur des Sciences de la Population, Ouagadougou, Burkina Faso; e International Centre for Reproductive Health—Kenya, Nairobi, Kenya; f Soins et Santé Primaire, CESP Centre for Research in Epidemiology and Population Health U1018, Inserm, Paris, France

## Abstract

**Background:**

Although hindrances to the sexual and reproductive health of women are expected because of COVID-19, the actual effect of the pandemic on contraceptive use and unintended pregnancy risk in women, particularly in sub-Saharan Africa, remains largely unknown. We aimed to examine population-level changes in the need for and use of contraception by women during the COVID-19 pandemic, determine if these changes differed by sociodemographic characteristics, and compare observed changes during the COVID-19 pandemic with trends in the 2 preceding years.

**Methods:**

In this study, we used four rounds of Performance Monitoring for Action (PMA) population-based survey data collected in four geographies: two at the country level (Burkina Faso and Kenya) and two at the subnational level (Kinshasa, Democratic Republic of the Congo and Lagos, Nigeria). These geographies were selected for this study as they completed surveys immediately before the onset of COVID-19 and implemented a follow-up specific to COVID-19. The first round comprised the baseline PMA panel survey implemented between November, 2019, and February, 2020 (referred to as baseline). The second round comprised telephone-based follow-up surveys between May 28 and July 20, 2020 (referred to as COVID-19 follow-up). The third and fourth rounds comprised two previous cross-sectional survey rounds implemented in the same geographies between 2017 and 2019.

**Findings:**

Our analyses were restricted to 7245 women in union (married or living with a partner, as if married) who were interviewed at baseline and COVID-19 follow-up. The proportion of women in need of contraception significantly increased in Lagos only, by 5·81 percentage points (from 74·5% to 80·3%). Contraceptive use among women in need increased significantly in the two rural geographies, with a 17·37 percentage point increase in rural Burkina Faso (30·7% to 48·1%) and a 7·35 percentage point increase in rural Kenya (71·6% to 78·9%). These overall trends mask several distinct patterns by sociodemographic group. Specifically, there was an increase in the need for contraception among nulliparous women across all geographies investigated.

**Interpretation:**

Our findings do not support the anticipated deleterious effect of COVID-19 on access to and use of contraceptive services by women in the earliest stages of the pandemic. Although these results are largely encouraging, we warn that these trends might not be sustainable throughout prolonged economic hardship and service disruptions.

**Funding:**

Bill & Melinda Gates Foundation.

**Translation:**

For the French translation of the abstract see Supplementary Materials section.

## Introduction

Sexual and reproductive health experts have predicted devastating consequences of the COVID-19 pandemic on the health of women.^1^, ^2^ Gender disparities are expected to be exacerbated by COVID-19^3^, ^4^ due to restricted access to health services, increased risk of gender-based violence, and severe economic effects.^4^, ^5^, ^6^ These forecasts draw heavily on data from previous epidemics, most notably, the 2016 Ebola outbreak in west Africa, which resulted in a substantial decline in family planning services (65% in Liberia; 23% in Sierra Leone).^7^ Although the effect of COVID-19 on sexual and reproductive health is largely unknown, global projections suggest the 2020 expected satisfied need for modern contraception could drop from 77% to 71% because of 60 million fewer contraceptive users.^8^

Adverse effects of the COVID-19 pandemic on the sexual and reproductive health of women are expected to be most prominent in low-income and middle-income countries (LMICs), which already face a multitude of challenges related to sexual and reproductive health service delivery.^9^ One modelling exercise projects that 15 million additional unintended pregnancies, leading to 28 000 maternal deaths, would occur over the course of one year if COVID-19-related health-care service disruptions affected 10% of women in need of sexual and reproductive health services in LMICs.^10^ Given mobility restrictions,^11^ shortages of contraceptive commodities,^6^, ^12^ and fear of exposure to COVID-19 when seeking services,^10^ these projections might be an underestimate.

**Research in context**

**Evidence before this study**

Modelling projections and evidence from previous epidemics and local crises have been pivotal in estimating the potential deleterious impact of the COVID-19 pandemic on the health and wellbeing of women. The actual effect of the pandemic on the reproductive health of women remains largely unknown, particularly in sub-Saharan Africa, where early preventive measures were implemented to control virus spread. We searched PubMed on Oct 15, 2020, limiting our search to articles published since March 1, 2020, when social movement restrictions began throughout sub-Saharan Africa, with no language restrictions, and using the search terms “(COVID-19 OR coronavirus) AND (reproductive health) AND (Africa OR sub-Saharan Africa).” We identified several commentaries or projections on the effects of COVID-19 on reproductive health. We did not identify any publications that collected quantitative data to understand the effects of COVID-19 on the need for and use of contraception by women in need in sub-Saharan Africa.

**Added value of this study**

To our knowledge, this is the first population-level study to estimate the need for contraception and contraceptive use among women in sub-Saharan Africa during the COVID-19 pandemic and relative to the pre-COVID-19 period. Using the Performance Monitoring for Action platform, we conducted national telephone follow-up interviews in Burkina Faso, Democratic Republic of the Congo (Kinshasa only), Kenya, and Nigeria (Lagos only) and compared these with baseline interviews done immediately before the pandemic (between November, 2019, and February, 2020). We found that the need for contraception increased significantly only in Lagos, and contraceptive use among women in need increased significantly in Kenya and rural Burkina Faso. These results highlight distinct subgroups that might be at increased risk for unintended pregnancies, namely, young women in Lagos and nulliparous women in Kinshasa.

**Implications of all the available evidence**

Our results are inconsistent with early modelling projections, which estimated significant decreases in contraceptive use, but should be interpreted with caution given the limitations of telephone follow-up and the early stage in the COVID-19 pandemic. Need for contraception and contraceptive use among women in need requires ongoing monitoring, particularly for the youngest and nulliparous women who might be at an increased risk of unintended pregnancy. Periods of prolonged economic hardship might further impede the ability of women to access and use contraception successfully.

Concerns surrounding the impact of COVID-19 on the sexual and reproductive health of women are particularly acute in sub-Saharan Africa. Although the region has achieved success in controlling SARS-CoV-2 spread via early lockdowns and restrictions on movement, these preventive measures took a toll on the African economy and health-care systems, potentially reversing recent progress in the area of sexual and reproductive health.^11^ Notably, before the onset of COVID-19, sub-Saharan Africa had an overall increase in modern contraceptive use;^13^ however, challenges in achieving universal access to sexual and reproductive health services, including family planning, remain substantial. In 2019, only 55% of sub-Saharan African women's need for contraception was satisfied and the unintended pregnancy rate (91 per 1000 women per year) was the highest globally.^14^

Early projections have been essential in raising awareness about the possible effects of COVID-19 on the sexual and reproductive health of women in sub-Saharan Africa and highlight the urgent need for evidence-based policies that balance prevention of COVID-19 transmission with access to sexual and reproductive health services. Few studies provide such timely information, and many rely on health systems data,^15^, ^16^ which do not necessarily reflect population-level changes in contraceptive use. Individual-level data from Burkina Faso and Kenya highlight little change in contraceptive use as a result of COVID-19;^17^ however, population-level data are needed to capture overall trends.

Across four sub-Saharan African geographies at various stages of the demographic transition and with diverse sociopolitical contexts, we aimed to examine population-level changes in the need for and use of contraception in women during the COVID-19 pandemic, determine if these changes differed by sociodemographic characteristics, and compare observed changes during COVID-19 with trends in the 2 years preceding the pandemic. We hypothesised that women's need for contraception would increase during the pandemic in relation to economic uncertainties. We anticipated that contraceptive use among women in need would decrease due to difficulties accessing services.

## Methods

### Study context

The first case of COVID-19 in Burkina Faso was reported on March 9, 2020, which triggered a national response operated by the Centre des Opérations de Réponse aux Urgences Sanitaires (Ouagadougou, Burkina Faso). Physical distancing measures successfully curbed the spread of the virus. Health services, including contraceptive services, remained open throughout the pandemic; however, fear of infection remained pervasive, prompting government-initiated radio messages informing the population of service availability. As of Feb 19, 2021, there were 11 703 confirmed cases and 139 deaths in Burkina Faso.^18^

In Democratic Republic of the Congo, the first case of COVID-19 was confirmed in Kinshasa on March 10, 2020. The president declared a state of emergency and closed borders on March 23, 2020; the State of Emergency was lifted on July 22, 2020. Use of health services that were deemed non-essential was discouraged. As of Feb 19, 2021, the country registered 24 794 confirmed cases, including 695 deaths; most cases have been concentrated in Kinshasa.^18^

The first case of COVID-19 in Kenya was reported on March 12, 2020. Since then, borders remain closed, gatherings are limited to less than 15 people (except for religious gatherings), and curfews are in place. Health services remained open, although shifted to focus on COVID-19 prevention and response. As of Feb 21, 2021, there were 103 841 confirmed cases and 1813 deaths.^18^

Lagos state has been the epicentre of the COVID-19 pandemic in Nigeria. During the first month of the pandemic (the first state lockdown began on March 23, 2020), non-essential government health services, including family planning services, closed completely in Lagos. Many parts of Lagos are densely populated, making it difficult for people to observe the recommended physical distancing guidelines. As of Feb 19, 2021, Lagos recorded 53 998 laboratory-confirmed cases and 381 deaths.

### Data source and study populations

This study draws on data from Performance Monitoring for Action (PMA), which conducts annual representative panel surveys on key reproductive health indicators in nine countries and regions in sub-Saharan Africa and southeast Asia. Further details on PMA methodology are available online.^19^ This analysis uses four rounds of PMA data, collected in four geographies: two at the country level (Burkina Faso and Kenya) and two at the subnational level (Kinshasa, Democratic Republic of the Congo and Lagos, Nigeria; table 1). These geographies were selected for this study as they completed surveys immediately before the onset of COVID-19 and implemented a follow-up specific to COVID-19.

**Table 1:** Data sources by data collection period and site

	**Year**	**Months**	**n**	**Response rate**
**Burkina Faso**				
PMA 2017	2017–18	November to January	3512	97·8%
PMA 2018	2018–19	December to January	3329	97·7%
Baseline	2019–20	December to February	6592	95·8%
COVID-19	2020	June to July	3518	57·7%
**Kinshasa, Democratic Republic of the Congo**				
PMA 2017	2017	September to October	2568	95·4%
PMA 2018	2018	October to December	2583	95·7%
Baseline	2019–20	December to February	2611	95·2%
COVID-19	2020	May to July	1312	68·1%
**Kenya**				
PMA 2017	2017	November to December	5876	99·0%
PMA 2018	2018	November	5671	99·1%
Baseline	2019	November to December	9474	98·7%
COVID-19	2020	May to July	5972	72·7%
**Lagos, Nigeria**				
PMA 2017	2017	January to May	1535	96·8%
PMA 2018	2018	April to May	1590	95·3%
Baseline	2019–20	December to January	1469	95·5%
COVID-19	2020	May to July	948	83·3%

PMA data for Democratic Republic of the Congo and Nigeria samples are specific to only Kinshasa and Lagos, respectively. PMA=Performance Monitoring for Action.

The first round comprised the baseline PMA panel survey implemented between November, 2019, and February, 2020 (referred to as baseline). At baseline, women responded to a face-to-face questionnaire soliciting information on their reproductive histories and behaviours and provided consent and contact information to participate in follow-up surveys.

The second round comprised telephone-based follow-up surveys between May 28 and July 20, 2020 (referred to as COVID-19 follow-up). These surveys were developed in response to COVID-19 to understand COVID-19-specific behaviours and reproductive health needs during the pandemic. Data were collected by remotely trained interviewers among the subsample of women who provided a telephone number and consent to be recontacted (response rate 57·7–83·3%).

The third and fourth rounds comprised two previous cross-sectional survey rounds implemented in the same geographies between 2017 and 2019 (referred to herein by country and survey year). These rounds used the same measures to assess sociodemographic characteristics and contraceptive behaviours as the baseline survey, facilitating the monitoring of national and regional trends in contraceptive indicators before the onset of COVID-19.

PMA2020 survey rounds, PMA baseline, and COVID-19 surveys received approval from ethical committees in each geography (Burkina Faso: Comité d'Ethique Institutionnel Pour La Recherche en Santé, Ministère de l'Enseignement Supérieur, de la Recherche Scientifique et de l'Innovation; Democratic Republic of the Congo: University of Kinshasa School of Public Health; Kenya: Kenyatta National Hospital—University of Nairobi Ethics Research Committee; Nigeria: Lagos State University Teaching Hospital Health Research Ethics Committee). The COVID-19 survey was also approved by the Johns Hopkins Bloomberg School of Public Health Institutional Review Board.

### Analytic samples

The baseline sample was restricted to women who responded to the COVID-19 survey to ensure the comparison of contraceptive indicators reflected a change in behaviours rather than a difference in sample selection. To confirm that the restricted baseline sample replicated characteristics of the full baseline sample, we assessed characteristics of women who were in union (married or living with a partner as if married) at baseline (full baseline survey), applying weights that accounted for the complex baseline survey design. Next, we compared characteristics of the full baseline sample with those of the restricted sample of women (in union, responded to the COVID-19 follow-up survey, and were successfully linked to their baseline interview data), accounting for baseline sampling weights. Finally, we compared the characteristics of the restricted sample to the full baseline sample of women in union, reweighting the restricted sample to account for loss to follow-up. Loss to follow-up weights were computed based on a woman's likelihood of owning a telephone and responding to the telephone survey as a function of their education, wealth, and residence. After adjusting for loss to follow-up, the restricted sample roughly mirrored the distribution of women's sociodemographic characteristics and contraceptive behaviours in the full baseline sample of women in union (appendix 2 p 1).

Comparative analyses to examine previous trends were done among the 2017 and 2018 PMA2020 samples of women in union.

### Measures

We examined two outcomes related to risk of unintended pregnancy measured before and during the COVID-19 pandemic: need for contraception and use of contraception among women in need. Women with a potential need for contraception were defined as those who ever had sexual intercourse, were married or in union (as a proxy for recent sexual activity in the absence of this information), not pregnant, not infertile, and did not intend to give birth in the next 12 months. Contraceptive use was defined as any use of contraception, regardless of the method used.

We considered a number of sociodemographic subgroups, by which we stratified estimates for our two outcomes, including age (15–24 years, 25–34 years, and 35–49 years), parity (nulliparous, 1–2 children, and ≥3 children), education (less than secondary or secondary or higher), household wealth (low, middle, or high), and household income loss during the COVID-19 pandemic (none, partial, or complete). All analyses for Burkina Faso and Kenya were stratified by residence (Lagos and Kinshasa were urban samples only) given recognised differences in fertility and use of sexual and reproductive health services in urban versus rural areas,^20^ as well as potential distinctions in the spread and impact of COVID-19 between settings.

### Analyses

This descriptive analysis first examined characteristics of women who participated in the COVID-19 follow-up survey by geography. Next, we estimated the proportion of women in need of contraception and the proportion of women using contraception among those in need at baseline and at the time of the COVID-19 follow-up survey within geographies and by sociodemographic characteristics. We estimated the changes in women's need for and use of contraception that occurred since COVID-19 by comparing point estimates for each outcome and sociodemographic subgroup between baseline and the COVID-19 follow-up survey. Significant changes in outcomes between timepoints were interpreted in relation to the CIs of the difference in the point estimates.

Finally, we contextualised findings with previous years' data by assessing trends in women's need for contraception and use of contraception among those in need across the four survey rounds. Each indicator was graphed over time per geography to disentangle the effect of the COVID-19 pandemic from existing changes occurring at the population level.

All analyses were done in STATA version 16 with statistical significance set at p<0·05. Complete case analysis was used, as missingness was less than 1%. Analyses were weighted to account for the complex survey design, including clustered sampling of women, non-response, and loss to follow-up in the COVID-19 pandemic.

### Role of the funding source

The funder of the study had no role in study design, data collection, data analysis, data interpretation, or writing of the report.

## Results

Our analyses were restricted to 7245 women in union who were interviewed at baseline and COVID-19 follow-up. Characteristics of the analytic sample are presented in table 2. The mean age of women was 31·8 years (SD 8·3) in Burkina Faso, 34·6 years (7·4) in Kinshasa, 32·6 years (7·7) in Kenya, and 35·9 years (6·4) in Lagos. Women's educational attainment differed substantially across geographies. Most women reported a partial loss of household income during the COVID-19 pandemic; complete loss of income was most common in Kinshasa. Need for contraception and contraceptive use by those in need varied across the geographies we studied.

**Table 2:** Characteristics of women at COVID-19 follow-up survey by site, weighted

		**Burkina Faso (n=2242)**	**Kinshasa, Democratic Republic of the Congo (n=560)**	**Kenya (n=3870)**	**Lagos, Nigeria (n=573)**
Residence					
	Urban	374 (16·7%)	560 (100·0%)	1082 (28·0%)	573 (100·0%)
	Rural	1868 (83·3%)	..	2788 (72·0%)	..
Age (years)					
	15–24	500 (22·3%)	50 (8·9%)	638 (16·5%)	15 (2·6%)
	25–34	905 (40·4%)	240 (42·9%)	1731 (44·7%)	229 (40·0%)
	35–49	837 (37·3%)	270 (48·2%)	1501 (38·8%)	329 (57·4%)
Parity					
	0	118 (5·3%)	41 (7·3%)	162 (4·2%)	29 (5·1%)
	1–2	678 (30·2%)	194 (34·7%)	1315 (34·0%)	228 (39·8%)
	≥3	1445 (64·5%)	325 (58·0%)	2393 (61·8%)	316 (55·1%)
Education					
	Less than secondary	2002 (89·4%)	61 (10·9%)	2249 (58·1%)	85 (14·8%)
	Secondary or higher	238 (10·6%)	499 (89·1%)	1621 (41·9%)	488 (85·2%)
Wealth					
	Low	813 (36·3%)	177 (31·6%)	1512 (39·1%)	179 (31·2%)
	Middle	853 (38·0%)	174 (31·1%)	1261 (32·6%)	179 (31·2%)
	High	576 (25·7%)	209 (37·3%)	1097 (28·3%)	215 (37·5%)
Economic loss due to COVID-19					
	None	514 (23·0%)	32 (5·8%)	242 (6·2%	31 (5·4%)
	Partial	1331 (59·4%)	153 (27·4%)	1962 (50·8%)	346 (60·4%)
	Complete	396 (17·7%)	373 (66·8%)	1664 (43·0%)	196 (34·2%)
Need for contraception		1569 (70·0%)	390 (69·6%)	3196 (82·6%)	460 (80·3%)
Contraceptive use among women in need		757 (50·9%)	263 (69·0%)	2574 (80·5%)	280 (61·6%)

Data are n (%). Due to small amounts of missing data, not all data sum to the totals in the table headings.

Between baseline and COVID-19 follow-up surveys, the proportion of women in need of contraception increased in all areas studied, although changes were only significant in Lagos (table 3; appendix 2 pp 2–3). Contraceptive use among women in need increased significantly in the two rural geographies, with a 17·37 percentage point increase in rural Burkina Faso (30·7% to 48·1%) and a 7·35 percentage point increase in rural Kenya (71·6% to 78·9%). Increases in contraceptive use in Kenya were similar between women in urban and rural areas (5·24 and 7·35 percentage points, respectively). No differences in contraceptive use were noted in other urban settings.

**Table 3:** Percentage point change in the proportion of women in need of contraception and the proportion of women using contraception among those in need before and during COVID-19

		**Burkina Faso**				**Kinshasa, Democratic Republic of the Congo**		**Kenya**				**Lagos, Nigeria**	
		Urban		Rural		Urban		Urban		Rural		Urban	
		Change	95% CI	Change	95% CI	Change	95% CI	Change	95% CI	Change	95% CI	Change	95% CI
**Total sample**													
In need		0·34	−3·64 to 4·32	1·27	−4·43 to 6·98	2·67	−3·85 to 9·18	3·48	−0·76 to 7·72	0·06	−2·74 to 2·87	5·81	1·29 to 10·34
Contraceptive use		2·79	−2·11 to 7·69	17·37	7·87 to 26·87	0·20	−8·03 to 7·63	5·24	1·21 to 9·27	7·35	5·10 to 9·59	−0·95	−8·33 to 6·43
**Age**													
Youngest (<25 years)													
	In need	6·05	−1·50 to 13·61	6·69	−8·48 to 21·87	14·02	−7·28 to 35·33	5·05	−4·15 to 14·25	6·62	−7·60 to 20·83	23·58	3·13 to 50·29
	Contraceptive use	6·14	−4·71 to 16·99	19·94	4·63 to 35·25	3·60	−21·36 to 28·57	3·90	−5·50 to 13·30	7·06	−0·46 to 13·67	−24·35	−56·54 to 7·85
Middle (25–34 years)													
	In need	0·12	−5·02 to 5·26	−4·60	−13·76 to 4·55	0·32	−8·82 to 9·46	2·91	−2·61 to 8·43	0·99	−2·85 to 4·83	9·95	1·56 to 18·34
	Contraceptive use	−1·32	−7·75 to 5·10	18·54	5·84 to 31·23	−4·61	−14·71 to 5·49	3·74	−1·38 to 8·86	4·73	−0·41 to 9·87	−2·36	−13·77 to 9·05
Oldest (≥35 years)													
	In need	−2·49	−8·69 to 3·70	4·28	−5·23 to 13·79	2·64	−6·30 to 11·59	3·47	−2·86 to 9·81	−3·48	−7·35 to 0·39	2·13	−3·82 to 8·08
	Contraceptive use	5·96	−2·55 to 14·47	15·05	1·58 to 28·52	3·24	−7·88 to 14·35	7·96	1·10 to 14·81	9·50	3·35 to 15·65	0·88	−9·18 to 10·94
**Parity**													
Nulliparous (parity=0)													
	In need	23·34	13·55 to 33·13	38·88	21·59 to 56·18	28·84	8·33 to 49·35	35·27	16·42 to 54·12	35·07	18·89 to 51·24	26·19	10·17 to 42·21
	Contraceptive use	26·30	1·26 to 51·35	−3·59	−63·85 to 56·67	−25·21	−77·13 to 26·70	2·09	−25·01 to 29·18	45·33	17·95 to 72·70	..	..
Low parity (1–2 children)													
	In need	−0·51	−6·34 to 5·31	1·56	−11·02 to 14·14	1·48	−8·13 to 11·09	0·80	−4·52 to 6·12	2·46	−2·61 to 7·53	6·82	−2·98 to 16·61
	Contraceptive use	2·01	−4·86 to 8·89	18·99	3·73 to 34·24	0·60	−11·96 to 13·17	5·38	−0·09 to 10·84	6·24	0·80 to 11·68	−2·08	−13·81 to 9·65
Higher parity (≥3 children)													
	In need	−2·54	−7·58 to 2·50	−1·49	−8·14 to 5·17	0·11	−8·20 to 8·42	1·90	−3·57 to 7·38	−2·70	−5·52 to 0·11	3·21	−1·23 to 7·65
	Contraceptive use	4·49	−1·88 to 10·86	18·00	7·80 to 28·21	1·96	−7·50 to 11·43	6·71	0·74 to 12·68	7·53	3·07 to 11·99	0·49	−9·22 to 10·21
**Education**													
Lower education (less than secondary)													
	In need	−1·72	−6·80 to 3·35	1·26	−4·75 to 7·28	12·60	−11·35 to 36·54	1·79	−4·36 to 7·93	−1·84	−5·21 to 1·54	3·84	−9·55 to 17·23
	Contraceptive use	2·18	−4·13 to 8·49	17·83	7·98 to 27·69	−8·12	−32·17 to 15·94	5·89	−0·04 to 11·74	7·30	2·48 to 12·12	7·44	−13·63 to 28·51
Higher education (secondary or more)													
	In need	3·99	−1·76 to 9·74	1·39	−15·37 to 18·15	1·46	−4·85 to 7·77	4·67	−0·52 to 9·86	3·53	−0·59 to 7·66	6·17	1·27 to 11·07
	Contraceptive use	3·60	−2·39 to 9·59	9·16	−16·51 to 34·84	1·05	−7·22 to 9·33	4·74	−0·48 to 9·96	7·26	2·48 to 12·04	−2·69	−10·10 to 4·72
**Wealth**													
Lowest tertile													
	In need	17·19	−14·63 to 49·02	2·89	−6·88 to 12·65	−3·74	−15·78 to 8·30	2·86	−14·60 to 20·33	−1·70	−5·72 to 2·32	3·95	−4·74 to 12·63
	Contraceptive use	2·51	−26·33 to 31·36	22·16	7·66 to 36·66	−4·04	−20·26 to 12·18	10·98	−1·40 to 23·35	9·04	3·48 to 14·60	−2·89	−15·91 to 10·14
Middle tertile													
	In need	−1·57	−17·20 to 14·06	−1·53	−10·30 to 7·23	12·20	0·32 to 24·73	2·14	−5·28 to 9·56	0·92	−2·71 to 4·55	6·98	−2·02 to 15·98
	Contraceptive use	1·41	−17·75 to 20·57	13·94	0·54 to 27·35	4·12	−8·20 to 16·43	6·51	0·01 to 13·02	5·88	1·09 to 10·67	0·56	−12·51 to 13·64
Highest tertile													
	In need	−0·03	−3·97 to 3·90	5·51	−6·11 to 17·14	0·16	−7·59 to 7·90	4·14	−0·69 to 8·97	3·99	−2·51 to 10·49	6·40	−1·30 to 14·09
	Contraceptive use	2·88	−2·19 to 7·96	12·09	−7·85 to 32·02	−0·45	−12·83 to 11·93	3·74	−5·90 to 13·37	4·88	−2·06 to 11·83	−0·58	−11·12 to 9·95
**Economic loss related to COVID-19**													
None													
	In need	2·68	−6·42 to 11·79	1·11	−9·54 to 11·77	−9·28	−29·30 to 10·74	10·69	−4·87 to 26·25	−3·87	−14·34 to 6·59	−10·92	−37·24 to 15·40
	Contraceptive use	3·96	−4·97 to 12·89	14·41	−0·54 to 29·36	16·75	−10·57 to 44·07	2·66	−12·66 to 17·98	9·88	0·40 to 19·37	10·96	−22·66 to 44·58
Partial													
	In need	−1·41	−5·75 to 2·92	2·86	−4·19 to 9·90	7·11	−4·84 to 19·06	2·40	−2·64 to 7·44	−0·05	−3·61 to 3·52	10·48	3·87 to 17·09
	Contraceptive use	2·67	−3·58 to 8·91	20·66	10·62 to 30·70	−10·23	−24·19 to 3·74	6·00	0·94 to 11·05	6·04	1·11 to 10·96	−2·39	−11·96 to 7·18
Complete													
	In need	4·98	−3·45 to 13·40	−3·59	−19·07 to 11·88	1·89	−6·52 to 10·31	4·02	−2·43 to 10·47	0·79	−2·94 to 4·52	0·24	−8·05 to 8·53
	Contraceptive use	1·90	−10·44 to 14·23	9·88	−11·66 to 31·42	1·95	−7·25 to 11·14	4·54	−1·77 to 10·84	8·45	3·17 to 13·72	−0·25	−12·62 to 12·13

An inadequate sample size of nulliparous women in need of contraception in Lagos prohibited analysis of contraceptive use.

These overall trends mask several distinct patterns by sociodemographic groups (table 3). Specifically, there was an increase in the need for contraception among nulliparous women in all geographies studied. Nulliparous women in rural Burkina Faso and urban and rural Kenya had the greatest increases in need (38·88, 35·27, and 35·07 percentage points, respectively). Need for contraception also increased for women with higher education (6·17 percentage points) and those who experienced partial income loss due to COVID-19 (10·48 percentage points) in Lagos.

Contraceptive use among women in need rose for specific subpopulations during the pandemic (table 3). Much greater proportions of nulliparous women in rural Kenya and urban Burkina Faso were using contraception during the COVID-19 pandemic. Significant increases in contraceptive use were also observed for almost every sociodemographic subgroup among rural women in Kenya and Burkina Faso. Although similar increases were noted among some subgroups in urban Kenya, such as older women and those with more children, broader changes by sociodemographic characteristics were not consistently observed in other urban settings. We found an increase in contraceptive use among women who reported partial income loss due to the COVID-19 pandemic in rural Burkina Faso. In contrast to these patterns, we noted non-significant drops in contraceptive coverage among young women in Lagos and among nulliparous women in Kinshasa.

**Figure 1:**
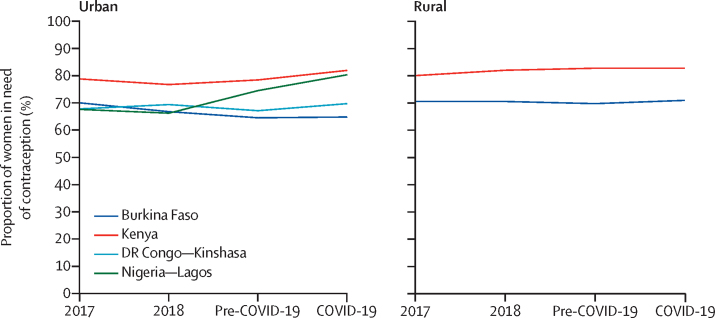
Changes in need for contraception from pre-COVID-19 to COVID-19 pandemic

Non-significant increases in the need for contraception during the COVID-19 pandemic (except in Lagos, where the increase was significant; figure 1) are consistent with trends observed in the year before the pandemic, except in urban Burkina Faso and Kinshasa, where such changes marked a departure from the previously observed decline.

**Figure 2:**
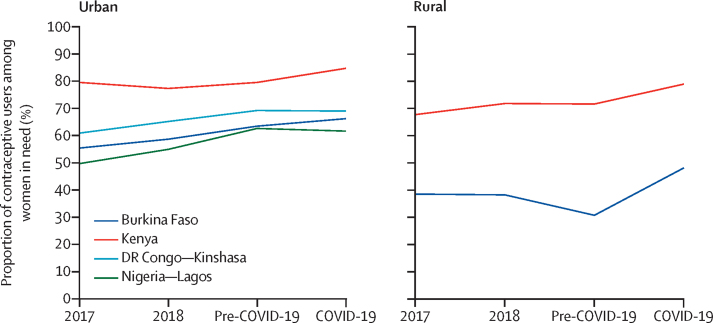
Changes in use of contraception among women in need from pre-COVID-19 to COVID-19 pandemic

The rise in contraceptive use during the COVID-19 pandemic followed a period of stagnation and decline in Kenya and rural Burkina Faso (figure 2). Conversely, in Kinshasa and Lagos, contraceptive use stabilised during the COVID-19 pandemic after years of substantial increases.

## Discussion

This analysis across four diverse geographies in sub-Saharan Africa does not support the anticipated deleterious effect of COVID-19 on women's access to and use of contraception in the earliest stages of the pandemic. Instead, we found that the COVID-19 pandemic was associated with marginal increases in women's need for contraception and larger increases in women's contraceptive use, although these trends vary by setting, women's characteristics, and reproductive life stage. Our results provide early evidence that the pandemic has so far, not altered the recent growth in contraceptive use, noted in several countries.^13^ Our results align with individual-level data indicating minimal shifts in contraceptive status in Kenya and Burkina Faso.^17^

Stratified analyses by residence revealed discernable differences in key contraceptive indicators. In contrast to our initial hypothesis, we found an increase in contraceptive use in the COVID-19 period in Kenya and rural Burkina Faso, but similar rises were not observed in Kinshasa or Lagos, where previous progress in meeting women's demand for contraception seemed to have stalled. Increases in use of contraception occurred in settings where women in union were more likely to rely on longer-acting contraceptive methods before the pandemic, including in rural Burkina Faso and Kenya, where 55% and 48% of contraceptive users, respectively, were using a long-acting method pre-pandemic. However, no increases in contraceptive use were noted in settings with a lower reliance on these methods (Kinshasa and Lagos). These findings could reflect an important benefit of long-acting methods in reducing unmet need for contraception by sustaining use,^21^ even in the face of service disruptions and potential commodity shortage. However, the substantial increase in contraceptive prevalence in rural areas also suggests fewer disruptions to sexual and reproductive health services in these settings than have been noted in facility-based studies in Ethiopia and South Africa.^15^, ^16^ The variability in contraceptive trends across geographies at the early stages of the COVID-19 pandemic in sub-Saharan Africa might also reflect differences in health system preparedness, as some countries, such as Burkina Faso, acted to prevent shortages by accruing large stocks of contraceptives.^22^ A thorough understanding of site-specific pandemic responses is crucial for monitoring sexual and reproductive health indicators in the region, as the effect of COVID-19 restrictions on women's access to contraception might be delayed, with areas requiring urgent interventions not yet apparent.

Furthermore, some differences in sexual and reproductive health indicators were observed by COVID-19-related economic losses, particularly in rural areas. Changes in contraceptive use did not accompany COVID-19-related income loss in Kinshasa, where 67% of women reported complete income loss. These descriptive results confirm the emerging concern with the impact of the COVID-19 pandemic on economic insecurity in sub-Saharan Africa.^23^ Economic concerns have been linked to changes in fertility intentions, as evidenced by sanitary crises^24^ and economic downturns.^25^ Similarly, we found increases in use of contraception among women who had partial economic losses in Kenya and rural Burkina Faso. Such changes might reflect stronger intentions to postpone or limit childbearing^26^ in the COVID-19 context among women who are able to access sexual and reproductive health care, whereas those unable to afford such services might benefit from targeted outreach programmes and free contraceptive provision.

Our results show further differences in women's sexual and reproductive health behaviours during the COVID-19 pandemic, according to their reproductive life stage and socioeconomic circumstances. An increase in the need for contraception was found among nulliparous women across all geographies studied. This finding might signal a desire to delay a first birth in the early stages of the pandemic, although the levels of need for contraception remained low among nulliparous women in all geographies studued. In urban Burkina Faso and rural Kenya, more women were able to act on their fertility preferences to prevent or delay pregnancy by using contraception. Importantly, this increase, albeit modest, occurred despite substantial normative barriers that discourage contraceptive use for women who still want children.^27^, ^28^ However, our results also indicate that these trends in contraceptive use are context-dependent, given that young women in Lagos and nulliparous women in Kinshasa had a drop in contraceptive coverage (albeit non-significant given large CIs). Exploration of motivation for this decline is warranted, as young women who are more likely to rely on coital-dependent methods might have less need for contraception if sexual activity patterns have changed, or might face greater access barriers due to the closures of government youth-friendly services.

This study is not without limitations. Foremost, telephone-based data collection was implemented given COVID-19 safety concerns, resulting in substantial sample restrictions (36·7–42·3% of the initial cohort excluded). Although post-stratification weights limit sample distortion, it is possible that sample selection could bias our COVID-19 survey estimates. Specifically, previous data collected in Burkina Faso indicate substantial differences in contraceptive use estimates between telephone and face-to-face interviews.^29^ Our results might similarly face such biases because of survey modality, as evidenced by large increases in contraceptive use. Furthermore, to minimise survey length, information about sexual activity was unavailable in the COVID-19 follow-up survey, which led to the exclusion of non-cohabiting partnered women from this analysis. The lack of data on women's sexual activity might have also impacted our estimates of women's need for contraception, as we assumed that women who were in union at baseline were and remained sexually active between surveys. Additionally, these analyses focus solely on women's contraceptive use, although we recognise the importance of couple reproductive decision making. Lastly, analyses were limited by small subgroup sample sizes, rendering large CIs, and preventing analysis for some subgroups (ie, nulliparous and young women).

Although these data were collected in the first few months of the COVID-19 pandemic, the combination of trends in need for and use of contraception could help policy makers understand potential trends in unintended pregnancy that might arise. Overall rates of unintended pregnancy might decline in rural settings of Burkina Faso and in Kenya, given substantial uptake of contraception. Our findings also identify subgroups of women who might have an increased risk of unintended pregnancy due to potential decreases in contraceptive use, specifically young women in Lagos and nulliparous women in Kinshasa.

In conclusion, our results provide early insights into the need for and use of contraception after restrictive COVID-19 lockdown measures across four settings in sub-Saharan Africa. Data suggest a minimal population-level effect on contraceptive coverage despite sustained demand for contraception, although trends vary by geography. Greater contraceptive coverage among women in need was observed in Kenya and rural settings in Burkina Faso, whereas younger women in Lagos and nulliparous women in Kinshasa might face heightened risks of unintended pregnancy related to declines in contraceptive coverage (albeit non-significant). Sustained use of contraception in most settings might reflect the fact that there were no contraceptive shortages (as commodity stocks had not run out yet) or that the effect of shortages was mitigated because of a high proportion of users relying on methods with prolonged duration of use (eg, injectables, implants, intrauterine devices, or multi-pack contraceptive pills). Although results from Kenya and Burkina Faso are encouraging, we warn against assumptions that women are protected from the future risk of unintended pregnancy, as our reported trends might not be sustainable throughout prolonged economic hardship and service disruption. As the health crisis turns into an economic crisis, we anticipate both the demand for contraception and supply of contraceptives will shift with decreasing levels of satisfied demand for contraception, resulting in increased unintended pregnancies.

## Data sharing

Data are available by request at pmadata.org.

## Declaration of interests

All authors report grants from the Bill & Melinda Gates Foundation during the conduct of the study.

## Supplementary Materials

French translation of the abstract
