# Supplementary material for: Need for and use of contraception by women before and during COVID-19 in four sub-Saharan African geographies: results from population-based national or regional cohort surveys
Source: Lancet Glob Health. 2021 May 18;9(6):e793–801. doi: 10.1016/S2214-109X(21)00105-4 (PMC8149322; doi:10.1016/S2214-109X(21)00105-4)
Supplement: French translation of the abstract [file mmc1.pdf]

THE LANCET
Global Health

Supplementary appendix 1

This translation in French was submitted by the authors and we reproduce it as supplied. It has not been peer reviewed. *The Lancet*'s editorial processes have only been applied to the original in English, which should serve as reference for this manuscript.

Cette traduction en français a été proposée par les auteurs et nous l'avons reproduite telle quelle. Elle n'a pas été examinée par des pairs. Les processus éditoriaux du *Lancet* n'ont été appliqués qu'à l'original en anglais et c'est cette version qui doit servir de référence pour ce manuscrit.

Supplement to: Wood SN, Karp C, OlaOlorun F, et al. Need for and use of contraception by women before and during COVID-19 in four sub-Saharan African geographies: results from population-based national or regional cohort surveys. *Lancet Glob Health* 2021; **9:** e793–801.

ABSTRACT

Contexte : Si l'on s'attend à des répercussions de la COVID-19 sur la santé sexuelle et reproductive des femmes, les conséquences réelles de la pandémie sur les pratiques contraceptives et les risques de grossesses non désirées, restent largement méconnues, en particulier en Afrique subsaharienne. Cette étude visait à estimer les changements en matière de besoins contraceptifs et d'utilisation de la contraception pendant la COVID-19, au niveau de la population; à déterminer si ces changements différaient selon les caractéristiques sociodémographiques des femmes ; et à comparer les changements observés pendant la COVID-19 avec les tendances des deux années précédentes.

Méthodes : Nous avons utilisé quatre séries de données d'enquête en population générale conduite par Performance Monitoring for Action (PMA) dans quatre géographies : deux au niveau national (Burkina Faso et Kenya) et deux au niveau régional (Kinshasa en République Démocratique du Congo et Lagos au Nigeria). Ces localités ont été choisies car elles disposaient de données collectées juste avant le début de l'épidémie ainsi qu'un suivi réalisé pendant l'épidémie. La première série correspond à la première vague des cohortes PMA collectées entre Novembre 2019 et Février 2020 (bases de référence). La deuxième série correspond à une enquête de suivi téléphonique COVID – collectée entre le 28 Mai et le 20 Juillet 2020 (enquêtes de suivi COVID). Les troisièmes et quatrièmes séries de données correspondent à deux enquêtes transversales réalisée dans les mêmes localités entre 2017 et 2019.

Résultats : Nos analyses portaient sur les 7216 femmes en union (mariées ou cohabitantes) interrogées lors de la première vague et lors du suivi téléphonique COVID-19. La proportion de femmes ayant des besoins contraceptifs a augmenté seulement à Lagos, passant de 74.5% à 80.3%, soit une progression de 5.81 points. Parmi les femmes ayant un besoin contraceptif, l'utilisation de la contraception a augmenté de manière significative dans les deux zones rurales de l'étude, avec une progression de 17.37 points au Burkina Faso (30·7% à 48·1%) et de 7.35 points au Kenya (71.6% à 78.9%). Les tendances générales masquent des propensions différentes selon les groupes sociodémographiques. En particulier, on note une augmentation des besoin contraceptifs chez les femmes nullipares dans tous les localités étudiées.

Interprétation : Nos résultats ne confirment pas l'effet délétère attendu de la COVID-19 sur l'accès et l'utilisation des services de contraception dans les premiers mois de la pandémie. Si ces résultats sont rassurants, la situation peut se dégrader au regard des difficultés économiques et des perturbations des services de santé qui se prolongent dans le temps.

Financement : Fondation Bill et Melinda Gates.
