# Supplementary material for: Need for and use of contraception by women before and during COVID-19 in four sub-Saharan African geographies: results from population-based national or regional cohort surveys
Source: Lancet Glob Health. 2021 May 18;9(6):e793–801. doi: 10.1016/S2214-109X(21)00105-4 (PMC8149322; doi:10.1016/S2214-109X(21)00105-4)
Supplement: Supplementary appendix 2 [file mmc2.pdf]

THE LANCET
Global Health

Supplementary appendix 2

This appendix formed part of the original submission and has been peer reviewed. We post it as supplied by the authors.

Supplement to: Wood SN, Karp C, OlaOlorun F, et al. Need for and use of contraception by women before and during COVID-19 in four sub-Saharan African geographies: results from population-based national or regional cohort surveys. *Lancet Glob Health* 2021; **9:** e793–801.

Appendix table 1. Loss to follow-up weighting

	Burkina Faso			DRC-Kinshasha			Kenya			Nigeria-Lagos		
	Baseline	COVID		Baseline	COVID		Baseline	COVID		Baseline	COVID	
		FQ·	LTFU		FQ·	LTFU·		FQ	LTFU		FQ	LTFU
	%											
Residence												
Urban	17·3	31·4	16·7	100·0	100·0	100·0	28·8	29·6	28·0	100	100	100·0
Rural	82·7	68·6	83·3	--	--	--	71·2	70·4	72·0	--	--	--
Age												
15-24	25·6	24·0	22·3	12·2	8·8	8·9	19·2	16·7	16·5	4·8	2·6	2·6
25-34	37·4	40·6	40·4	41·1	42·4	42·9	43·0	45·0	44·7	40·4	40·4	40·0
35-49	37·0	35·5	37·3	46·7	48·9	48·2	37·9	48·3	38·8	54·8	57·1	57·4
Parity												
0	6·9	6·6	5·3	7·9	8·2	7·3	5·3	4·4	4·2	6·5	5·3	5·1
1-2	29·1	34·3	30·2	35·1	35·2	34·7	35·7	35·5	34·0	41·8	40·5	39·8
3+	64·0	59·1	64·5	57·0	56·6	58·0	58·9	60·1	61·9	51·7	54·2	55·1
Education												
<Secondary	88·0	80·1	89·4	11·5	6·0	10·8	57·5	53·2	58·1	15·2	12·8	14·8
Secondary+	12·0	19·9	10·6	88·6	94·1	89·2	42·5	46·8	41·9	84·8	87·2	85·2
Need for contraception	71·6	67·2	68·9	68·1	65·4	67·1	80·8	80·6	81·6	70·9	73·5	74·5
Contraception among women in need	38·0	41·6	35·8	69·8	69·7	69·2	69·8	74·1	73·7	60·5	63·2	62·6

Baseline=married/currently partnered women at baseline survey weighted using female questionnaire weights
COVID FQ= married/currently partnered women at COVID survey weighted using female questionnaire weights
COVID LTFU=married/currently partnered women at COVID survey weighted (adjusted for selectivity due to phone ownership and loss-to-follow-up)

Appendix table 2. Point estimates for unmet need and contraceptive use among women in need (95% confidence interval)

	Burkina Faso				DRC-Kinshasa		Kenya				Nigeria-Lagos	
	Urban		Rural		Urban		Urban		Rural		Urban	
	Baseline	COVID	Baseline	COVID	Baseline	COVID	Baseline	COVID	Baseline	COVID	Baseline	COVID
**Total Sample**												
In need	64·5 (61·6, 67·2)	64·8 (61·8, 67·7)	69·8 (64·8, 74·3)	71·0 (67·5, 74·3)	67·1 (62·1, 71·2)	69·7 (64·9, 74·2)	78·4 (75·0, 81·6)	81·9 (79·0, 84·5)	82·8 (80·8, 84·6)	82·8 (80·7, 84·8)	74·5 (71·1, 77·6)	80·3 (76·8, 83·3)
Contraceptive use	63·4 (59·7, 66·9)	66·2 (62·7, 69·5)	30·7 (24·7, 37·4)	48·1 (40·9, 55·3)	69·2 (62·8, 75·0)	69·0 (63·6, 73·9)	79·5 (76·0, 82·5)	84·7 (82·1, 87·0)	71·6 (68·8, 74·2)	78·9 (76·0, 81·5)	62·6 (56·8, 68·0)	61·6 (56·6, 66·4)
**Age**												
Youngest <25												
In need	57·2 (52·1, 62·2)	63·3 (57·4, 68·8)	64·9 (51·9, 76·0)	71·6 (61·2, 80·1)	64·3 (47·5, 78·1)	78·3 (59·2, 90·0)	73·1 (65·8, 79·3)	78·1 (71·1, 83·9)	71·5 (65·9, 76·4)	78·1 (72·2, 83·0)	69·6 (41·1, 88·3)	93·2 (59·0, 99·2)
Contraceptive use	54·5 (46·2, 62·5)	60·6 (53·1, 67·7)	22·5 (13·8, 34·5)	42·4 (31·3, 54·5)	69·5 (44·1, 86·8)	73·1 (57·8, 84·3)	80·2 (72·7, 86·0)	84·1 (76·0, 89·8)	78·9 (73·3, 83·6)	86·0 (81·2, 89·7)	82·8 (50·7, 95·8)	58·5 (30·9, 81·5)
Middle (25-34)												
In need	65·5 (61·9, 68·8)	65·6 (61·6, 69·3)	75·1 (67·2, 81·7)	70·5 (64·4, 76·0)	69·6 (62·0, 76·2)	69·9 (63·5, 75·6)	77·9 (73·5, 81·8)	80·9 (76·8, 84·3)	81·2 (78·3, 83·7)	82·1 (79·2, 84·7)	65·3 (58·8, 71·2)	75·2 (69·0, 80·5)
Contraceptive use	70·4 (65·4, 75·0)	69·1 (64·5, 73·3)	33·0 (25·4, 41·6)	51·5 (41·5, 61·5)	76·5 (70·0, 82·1)	71·9 (62·9, 79·4)	83·0 (78·7, 86·6)	86·7 (83·0, 89·7)	77·4 (73·5, 81·9)	82·1 (78·2, 85·5)	62·7 (53·4, 71·2)	60·4 52·9, 67·3)
**Oldest (35+)**												
In need	67·3 (62·8, 71·4)	64·8 (60·1, 69·2)	66·9 (58·4, 74·4)	71·2 (65·4, 76·3)	65·4 (59·4, 70·9)	68·0 (60·5, 74·6)	82·0 (76·3, 86·6)	85·5 (81·2, 89·0)	88·9 (85·9, 91·3)	85·4 (82·4, 88·0)	81·1 (76·5, 85·0)	83·2 (78·5, 87·1)
Contraceptive use	59·9 (54·2, 65·2)	65·8 (58·9, 72·1)	32·8 (24·9, 41·7)	47·8 (37·2, 58·6)	62·3 (53·7, 70·2)	65·5 (57·5, 72·8)	74·3 (68·8, 79·2)	82·3 (77·2, 86·4)	63·6 (59·1, 67·9)	73·1 (68·6, 77·2)	61·7 (53·8, 69·0)	62·6 (55·6, 69·0)
**Parity**												
**Nulliparous (parity=0**												
In need	7·5 (4·0, 13·6)	30·8 (22·8, 40·3)	4·4 (1·0, 17·8)	43·2 (29·1, 58·5)	16·6 (7·2, 34·0)	45·5 (29·8, 62·1)	25·6 (14·9, 40·3)	60·8 (45·9, 74·0)	18·6 (10·4, 30·9)	53·6 (40·7, 66·1)	4·4 (0·9, 18·3)	30·6 (17·5, 47·8)
Contraceptive use	10·0 (0·9, 58·1)	36·3 (22·0, 53·4)	22·6 (0·0, 99·7)	19·0 (4·7, 52·7)	53·2 (9·2, 92·7)	28·0 (9·6, 58·8)	54·6 (29·6, 77·4)	56·7 (35·7, 75·5)	20·8 (6·1, 51·5)	66·2 (46·6, 81·4)		
**Low parity (1-2 children)**												
In need	63·4 (58·8, 67·7)	62·9 (58·9, 66·7)	66·1 (57·2, 74·0)	67·6 (57·3, 76·5)	58·6 (51·3, 65·5)	60·1 (53·2, 66·6)	76·5 (72·5, 80·1)	77·3 (73·3, 80·9)	73·4 (69·5, 77·0)	75·9 (72·2, 79·2)	63·7 (55·8, 70·8)	70·5 (63·7, 76·5)
Contraceptive use	62·1 (57·3, 66·8)	64·2 (58·9, 69·1)	25·6 (16·2, 37·9)	44·5 (33·8, 55·8)	71·7 (61·2, 80·3)	72·3 (63·0, 80·0)	79·9 (75·5, 83·6)	85·3 (81·1, 88·6)	75·8 (71·5, 79·7)	82·0 (78·1, 85·4)	60·4 (51·3, 68·9)	58·3 (50·3, 66·0)
**Higher parity (3+ children)**												
In need	74·4 (71·0, 77·6)	71·9 (67·8, 75·6)	75·9 (70·5, 80·6)	74·4 (69·6, 78·7)	78·4 (72·2, 83·6)	78·5 (71·6, 84·3)	87·4 (82·6, 91·1)	89·3 (85·1, 92·5)	90·0 (88·0, 91·6)	87·3 (84·9, 89·3)	88·7 (85·1, 91·6)	91·9 (88·2, 94·6)
Contraceptive use	65·2 (60·5, 69·7)	69·7 (65·0, 74·0)	32·6 (26·2, 38·3)	50·6 (42·4, 58·7)	68·5 (60·6, 75·5)	70·5 (64·0, 76·2)	80·1 (75·0, 84·0)	86·8 (82·5, 90·2)	70·6 (65·7, 72·6)	78·1 (74·8, 81·2)	63·7 (55·9, 70·9)	64·2 (57·8, 70·2)
**Education**												
**Lower education (<secondary)**												
In need	64·3 (60·6, 67·8)	62·6 (58·9, 66·2)	69·8 (64·7, 74·5)	71·1 (67·3, 74·6)	68·4 (46·3, 84·5)	81·0 (60·0, 92·4)	81·3 (76·5, 85·3)	83·1 (78·2, 87·1)	85·3 (82·8, 87·4)	83·5 (80·8, 85·8)	84·7 (71·7, 92·4)	88·6 (75·5, 95·1)
Contraceptive use	58·1 (53·3, 62·9)	60·3 (56·1, 64·4)	29·3 (23·1, 36·4)	47·2 (39·8, 54·7)	63·8 (42·6, 80·7)	55·7 (39·0, 71·2)	78·4 (73·5, 82·7)	84·3 (80·2, 87·7)	70·1 (66·5, 73·5)	77·4 (73·9, 80·6)	47·9 (32·7, 63·5)	55·4 (40·6, 69·2)

**Higher education (>=secondary)**												
In need	64·4 (60·5, 68·1)	68·4 (63·9, 72·6)	68·3 (55·5, 78·8)	69·7 (55·9, 80·7)	66·9 (62·1, 71·4)	68·4 (63·8, 72·6)	76·4 (72·1, 80·3)	81·2 (77·6, 84·2)	78·1 (75·2, 80·9)	81·7 (78·5, 84·6)	72·6 (68·9, 76·1)	78·8 (75·2, 82·0)
Contraceptive use	72·2 (67·8, 76·1)	75·8 (71·1, 79·9)	54·3 (34·6, 72·8)	63·5 (45·0, 78·7)	69·9 (63·2, 75·9)	71·0 (65·1, 76·2)	80·3 (76·1, 83·9)	85·0 (81·0, 88·3)	74·4 (70·9, 77·6)	81·7 (78·0, 84·9)	65·4 (59·7, 70·7)	62·7 (57·6, 67·6)
**Wealth**												
**Lowest tertile**												
In need	56·0 (30·1, 79·0)	73·2 (43·4, 90·1)	68·5 (60·0, 75·8)	71·3 (64·9, 77·0)	75·3 (66·7, 82·3)	71·6 (60·1, 80·2)	80·3 (66·7, 89·2)	83·1 (64·1, 93·2)	83·1 (80·2, 85·7)	81·4 (78·3, 84·3)	78·9 (72·4, 84·2)	82·8 (75·2, 88·5)
Contraceptive use	67·7 (48·0, 82·7)	70·2 (39·0, 89·7)	30·8 (24·4, 40·6)	53·0 (41·2, 64·4)	68·8 (55·3, 79·8)	64·8 (52·9, 75·1)	76·3 (65·7, 84·4)	87·3 (75·7, 93·8)	69·4 (65·2, 73·2)	78·4 (74·2, 82·1)	60·0 (49·9, 69·4)	57·1 (48·1, 65·7)
**Middle tertile**												
In need	73·2 (60·1, 83·2)	71·6 (58·9, 81·6)	73·7 (65·9, 80·2)	72·2 (66·5, 77·2)	62·4 (51·7, 72·0)	74·6 (66·2, 81·5)	78·5 (72·1, 83·7)	80·6 (75·4, 85·0)	85·1 (82·4, 87·5)	86·0 (83·2, 88·5)	71·7 (65·4, 77·3)	78·7 (71·0, 84·8)
Contraceptive use	60·1 (45·5, 73·2)	61·5 (46·6, 74·5)	29·0 (19·4, 40·9)	43·0 (34·9, 51·4)	67·3 (57·7, 75·6)	71·4 (61·8, 79·4)	79·3 (73·7, 84·0)	85·8 (81·1, 89·5)	73·3 (70·0, 76·5)	79·2 (75·4, 82·5)	59·7 (49·6, 69·1)	60·3 (51·2, 68·7)
**Highest tertile**												
In need	64·0 (61·3, 66·7)	64·0 (61·0, 66·8)	60·6 (50·7, 69·7)	66·1 (58·7, 72·8)	64·0 (58·3, 69·2)	64·1 (58·1, 69·7)	78·1 (74·1, 81·6)	82·3 (78·9, 85·2)	75·9 (70·7, 80·5)	79·9 (75·2, 83·9)	73·1 (66·4, 78·9)	79·5 (74·4, 83·8)
Contraceptive use	63·6 (59·7, 67·3)	66·5 (63·0, 69·8)	37·3 (26·5, 49·6)	49·4 (33·2, 65·7)	71·2 (60·9, 79·7)	70·8 (61·6, 78·5)	80·1 (75·7, 83·9)	83·8 (80·7, 86·6)	74·9 (69·1, 80·0)	79·8 (75·1, 83·9)	67·2 (58·8, 74·6)	66·6 (59·1, 73·4)
**Economic loss related to COVID**												
**None**												
In need	65·6 (58·6, 71·9)	68·2 (61·6, 74·2)	70·8 (61·5, 78·6)	71·9 (64·7, 78·1)	84·8 (65·8, 94·2)	75·5 (56·3, 88·0)	72·2 (58·5, 82·7)	82·9 (70·0, 90·9)	80·5 (71·2, 87·3)	76·7 (69·0, 82·9)	73·5 (49·9, 88·5)	62·6 (42·3, 79·2)
Contraceptive use	60·3 (53·9, 66·4)	64·3 (57·5, 70·5)	25·4 (15·9, 37·9)	39·8 (29·8, 50·7)	62·2 (40·1, 80·1)	78·9 (53·2, 92·5)	82·1 (68·9, 90·6)	84·8 (69·6, 93·2)	76·6 (69·1, 82·8)	86·5 (78·2, 92·0)	56·1 (32·8, 77·1)	67·1 (37·1, 87·6)
**Partial**												
In need	65·0 (61·8, 68·1)	63·4 (60·5, 66·5)	69·8 (63·5, 75·4)	72·6 (68·4, 76·5)	60·5 (51·3, 69·0)	67·6 (58·7, 75·3)	79·9 (75·3, 83·8)	82·3 (79·3, 85·0)	82·6 (80·6, 84·5)	82·6 (79·4, 85·4)	72·8 (67·5, 77·5)	83·3 (78·4, 87·3)
Contraceptive use	64·1 (59·2, 68·6)	66·7 (62·4, 70·8)	31·9 (25·4, 39·2)	52·6 (45·0, 60·0)	73·6 (62·0, 82·7)	63·4 (53·1, 72·6)	79·9 (75·5, 83·6)	85·9 (82·4, 88·7)	72·3 (68·9, 75·4)	78·3 (74·3, 81·8)	64·0 (56·7, 70·8)	61·7 (54·9, 68·0)
**Complete**												
In need	60·9 (55·0, 66·5)	65·9 (59·3, 71·9)	68·4 (56·4, 78·3)	64·8 (52·5, 75·4)	68·1 (61·8, 73·8)	70·0 (63·5, 75·8)	77·3 (72·9, 81·1)	81·3 (75·6, 85·9)	83·3 (80·3, 85·9)	84·1 (81·4, 86·4)	77·6 (71·3, 82·7)	77·8 (71·0, 83·3)
Contraceptive use	64·5 (56·3, 71·9)	66·4 (56·0, 75·4)	33·9 (19·9, 51·5)	43·8 (29·4, 59·4)	68·2 (61·3, 74·4)	70·1 (63·0, 76·4)	78·7 (73·4, 83·1)	83·2 (78·6, 86·9)	70·1 (66·0, 73·9)	78·5 (74·8, 81·9)	61·1 (51·4, 70·0)	60·8 (52·3, 68·8)

Inadequate sample size of nulliparous women in need of contraception in Lagos prohibited analysis of contraceptive use
